# An HIV-1 Envelope Immunogen with W427S Mutation in CD4 Binding Site Induced More T Follicular Helper Memory Cells and Reduced Non-Specific Antibody Responses

**DOI:** 10.1371/journal.pone.0115047

**Published:** 2014-12-29

**Authors:** Hao-Tong Yu, Dan Tian, Jia-Ye Wang, Cai-Xia Guo, Yan Li, Xin Wang, Di Li, Feng-Min Zhang, Min Zhuang, Hong Ling

**Affiliations:** 1 Department of Microbiology, Harbin Medical University, Harbin, China; 2 Heilongjiang Provincial Key Lab for Infection and Immunity, Key Lab of Etiology of Heilongjiang Province Education Bureau, Harbin, China; 3 Department of Parasitology, Harbin Medical University, Harbin, China; Shanghai Medical College, Fudan University, China

## Abstract

The CD4 binding site (CD4BS) of the HIV-1 envelope glycoprotein (Env) contains epitopes for broadly neutralizing antibody (nAb) and is the target for the vaccine development. However, the CD4BS core including residues 425-430 overlaps the B cell superantigen site and may be related to B cell exhaustion in HIV-1 infection. Furthermore, production of nAb and high-affinity plasma cells needs germinal center reaction and the help of T follicular helper (Tfh) cells. We believe that strengthening the ability of Env CD4BS in inducing Tfh response and decreasing the effects of the superantigen are the strategies for eliciting nAb and development of HIV-1 vaccine. We constructed a gp120 mutant W427S of an HIV-1 primary R5 strain and examined its ability in the elicitation of Ab and the production of Tfh by immunization of BALB/c mice. We found that the trimeric wild-type gp120 can induce more non-specific antibody-secreting plasma cells, higher serum IgG secretion, and more Tfh cells by splenocyte. The modified W427S gp120 elicits higher levels of specific binding antibodies as well as nAbs though it produces less Tfh cells. Furthermore, higher Tfh cell frequency does not correlate to the specific binding Abs or nAbs indicating that the wild-type gp120 induced some non-specific Tfh that did not contribute to the production of specific Abs. This gp120 mutant led to more memory Tfh production, especially, the effector memory Tfh cells. Taken together, W427S gp120 could induce higher level of specific binding and neutralizing Ab production that may be associated with the reduction of non-specific Tfh but strengthening of the memory Tfh.

## Introduction

Designing an ideal immunogen that can elicit potently and broadly neutralizing antibodies (bnAbs) to primary virus isolates is a major challenge in developing a vaccine for human immunodeficiency virus type 1 (HIV-1) [1], [2]. Three clinical trials using HIV-1 envelope glycoprotein (Env) immunogens revealed that they did not show ideal protection [3]–[6]. The vaccine RV144 displayed only 31.2% protection against HIV-1 infection. But the protection efficacy correlated with the binding of IgG antibodies to variable regions 1 and 2 (V1/V2) of Env rather than neutralization effect though it induced weak nAb responses [7]–[9].

Since Env engages the cellular CD4 molecules and forms a CD4 binding site (CD4BS) on its surface [10]–[12], which is a highly conserved domain among various HIV-1 subtypes, CD4BS is considered as the main target for nAbs [13]–[16]. Recently, efforts to identify and characterize bnAbs from HIV-1 infected individuals have provided important insights into the molecular mechanisms of HIV-1 neutralization [17]–[21]. To date, four classes of anti-CD4BS bnAbs have been defined: b12, HJ16, VRC01, and 8ANC131 [22]. VRC01-like bnAbs have been isolated from several HIV-1 infected individuals and characterized [15], [16]. However, among HIV-1 infected individuals, only a small proportion develops bnAbs against CD4BS [15], [23]. Furthermore, despite the presence of anti-CD4BS epitopes on recombinant Envs, the immunization using such immunogens has failed to elicit such antibodies [24]–[29]. The reasons why anti-CD4BS bnAbs are rarely produced either by immunization or during natural HIV-1 infection are not well understood yet.

Extensive studies of germlines of bnAbs have revealed that the predicted germline precursors for VRC01-like bnAbs exhibit no detectable affinity for wild-type Env [16], [20]. This is a possible explanation for the rarity of VRC01-like bnAbs in HIV-1 infection. More importantly, wild-type Envs lacking germline affinity are poor to prime VRC01-like responses, because they are unlikely to reliably stimulate germline precursors to initiate antibody affinity maturation [30]. Therefore, despite the presence of broad anti-CD4BS neutralization, using this knowledge to rationally develop an effective immunogen continues to be difficult [31].

It has been accepted that affinity maturation of nAbs and high affinity plasma cells need germinal center (GC) reaction and the help of T follicular helper cells (Tfh) [16]. Recent studies have suggested that the control of HIV-1 infection progression in elite controllers is involved in the generation of bnAbs which undergo extensive affinity maturations in GCs [7], [32]. A subset of circulating CXCR5^+^CD4^+^ cells expressing programmed cell death protein 1 (PD-1), PD-1^+^CXCR3^-^CXCR5^+^CD4^+^ population, has been identified as the most closely resemble GC Tfh cells in peripheral blood and has memory-like phenotype. The ability of the HIV-infected individuals to develop bnAbs depends on the presence of this circulating Tfh, memory-like cells in the blood. Therefore, HIV-1 vaccine candidates that are designed to boost this subset of T cells would be promising prophylactic or therapeutic immunogens.

It has been found that during HIV chronic infection, both non-virus-specific and virus-specific Tfh cells are significantly expanded, but infected individuals rarely develop functional Tfh cells [33], [34]. Moreover, Tfh cell expansion has been also found in simian immunodeficiency virus (SIV) infections [35], [36]. The significance of the expansion of Tfh cells in developing nAbs during HIV-1 infection is still unknown and may not provide an insight into Env immunization. Very recently, Hollister K and coauthors found a phenomenon that though the immunization with HIV-1 gp120 induced Tfh cell differentiation, GC B cell responses and antigen-specific antibodies, GC reaction may actually limit antigen-specific IgG secretion in the context of repeated immunizations [37]. Apparently, the true Tfh cell functions in helping B cell differentiation into antibody-producing plasma cells and antibody production in the context of HIV-1 Env immunization are still needed to be studied extensively.

Moreover, key protective features of antibody responses including affinity maturation and B cell memory would be also necessary to be better understood. B cell memory occurs as a consequence of the ability of Tfh cells to initiate GC reactions [1], [7]. The interaction of T and B cells is initiated by a cognate antigen-primed B cells and T cells and provides co-stimulatory signals by interactions of ICOSL-ICOS, B7-CD28, CD40-CD40L and CD84-CD84 [35], [36]. After receiving help of antigen-specific T cells, B cells enter into extrafollicular regions to differentiate into short-lived plasma cells or expand rapidly to form GC [7]. GCs have been regarded as dedicated microenvironments in which somatic hypermutation (SHM) takes place to facilitate affinity maturation. The B cells expressing B cell antigen receptors (BCRs) with high affinity can be selected to terminally differentiate into long-lived memory B cells and memory plasma cells [1], [2]. In HIV-1 infected individuals, B cell dysregulation have been also found. The expansion of immature, pre-GC B cells, plasmablast, and plasma cells drives the secretion of non-specific polyclonal antibodies at high levels [38], [39]. The mechanisms are still not well elucidated.

It has been found that the CD4BS core including residues 425–430 overlaps a B cell superantigen site [40] and we investigated whether the modified Env with W427S, aborting CD4 binding but maintaining the binding ability to CD4BS Abs [12], could reduce interference of superantigen property. In the present study, we examined the ability of the Env gp120 with W427S of an HIV-1 primary R5 strain in Ab elicitation and in Tfh amount, function and memory in immunized BALB/c mice to try to clarify the Tfh responses and immune memory induced by Env immunization to facilitate the understanding about its weak immunogenicity.

## Materials and Methods

### Ethic Statement

The study was approved by the Harbin Medical University Ethics Committee (HMUIRB20140020). Female BALB/c mice (6-7 weeks) were purchased from Vital River Laboratories (Beijing) and housed in the Harbin Medical University Animal Laboratory. Mice were housed under standardized light-controlled conditions at room temperature (24°C) and 50% humidity, with free access to food and water. Animal experiments were performed in strict accordance with the recommendations in the Guide for the Care and Use of Laboratory Animals of the National Institutes of Health. All surgery was performed under sodium pentobarbital anesthesia, and all efforts were made to minimize suffering.

### Antigens and Adjuvants

Codon optimization of gp120 gene of HIV-1 primary isolate 06044 (EU131805) wild-type was performed according to the mammalian bias. Both gp120s containing trimeric motif and tissue plasminogen activator signal peptide can form stable trimer and secrete into supernatant [41]. The W427S mutant [12] was constructed using mutagenesis kit as described previously [41]. Plasmid DNA was prepared using Zyppy Plasmid Miniprep kit (Zymo research) and transient transfection of HEK293T cells was done using Lipofectamin 2000 reagents (Invitrogen). The supernatants were harvested at 60 h after transfection. The trimeric Env proteins were quantified on 8% native polyacrylamide gel and detected by western blot. The Env proteins in the gels were excised, grinded and then formulated with appropriate AddaVax adjuvants (Invivogen), and the mixtures were used as immunogens [42], [43].

### Immunizations

Female BALB/c (6-week old) were purchased from Vital River Laboratories (Beijing) and housed in the Harbin Medical University Animal Laboratory. All mice used in the experiments were treated according to the animal care committee guidelines of the Harbin Medical University. Five mice per group were immunized intramuscularly with DNA (40 µg per mouse) at weeks 0, 2 and 4, and subcutaneously with adjuvant-formulated Env proteins (15 µg per mouse) at weeks 8 and 11. At 14^th^ day post-final immunization, mice were sacrificed, and sera, splenocytes and bone marrow cells were collected to evaluate humoral and cellular immune responses.

### Detection of Serum Env-specific Abs and Serum IgG Concentrations

HIV-1 Env-specific antibodies in serum samples were analyzed using ELISA. Ninety-six-well plates (NUNC) were coated with HIV-1 Bal gp120 (The reagent was obtained through the NIH AIDS Reagent Program, Division of AIDS, NIAID, NIH: HIV-1_BaL_ gp120 from DAIDS, NIAID) or trimeric 06044 gp120 proteins [41] at a final concentration of 0.5 µg/ml at 4°C overnight. Then, the plates were blocked with PBST containing 1% BSA for 1 h at 37°C. After washing with PBST, serum samples at a 10-fold serial dilution (with a start dilution of 1∶100) were incubated in the coated plates for 2 h at 37°C. The plates were then washed three times and incubated with HRP-labeled goat anti-mouse IgG (ZSGB-BIO) at a dilution of 1∶1000 for 30 min at room temperature (RT). After washes, the substrates were added and the reactions were stopped with 2 M H_2_SO4. The optical density at 450 nm was measured using the Model 550 Microplate Reader (Bio-Rad).

Serum IgG concentrations were quantified using Mouse IgG total ELISA Ready-SET-Go ELISA Kit (eBioscience) following the manufacturer's instructions. Briefly, the capture antibodies were adsorbed onto 96-well plates overnight at 4°C. The plates were washed twice with PBST and then incubated with Blocking Buffer at RT for 2 h. The plates were washed twice with PBST, then the serum samples (1∶250) or standards (2-fold serial dilutions) with detection antibodies were added and incubated at RT for 3 h. After washing, substrates were added for 15 min followed by the addition of the stop solution. The optical density at 450 nm was measured using the Model 550 Microplate Reader (Bio-Rad).

### Neutralization Assay

Neutralizing activities were detected using HIV-1 SF162 or 06044 wild-type Env-pseudoviruses with TZM-bl cells as described previously [44]. BALB/c mouse sera were heat-inactivated at 56°C for 1 h prior to the assay. Briefly, 10 µl sera were diluted in 40 µl DMEM solution containing 10% heat-inactivated FBS, equal volumes (25 µl) of sera and SF162 pseudovirus (100 TCID50) were added to a 96-well plate in duplicates. After 1 h of incubation, 50 µl TZM-bl cell suspension including 10,000 cells was added to each well. The plates were incubated for 48 h, then 100 µl Bright-Glo Luciferase assay System (Promega) was added to each well and incubated at RT for 5 min. The relative luminescence unit (RLU) in each well was read with a ModulusII microplate multimode reader (Turner biosystems). The neutralization percentages were calculated as the percentage of reduction in RLU in wells containing pseudovirus and serum relative to the RLU in wells containing only pseudovirus after subtraction of background RLU in cell control wells.

### B Cell ELISPOT Assay

The B cell Elispot assay was performed as described previously using kit ELISpot ^PLUS^ for Mouse IgG (Mabtech) [45]. Briefly, 96-well MultiScreen-IP filter plate was pretreated with 70% ethanol and washed three times in PBS and coated with 10 µg/ml polyclonal goat anti-mouse IgG Ab, then the plate was incubated at 4°C overnight. After washes, the plate was blocked with complete RPMI 1640 medium at 37°C for 2 h, and appropriate numbers of BM cells (1×10^6^ for specific or 1×10^5^ for total IgG-secreting cell detection) were added to the wells in duplicates. The plate was incubated at 37°C overnight and added with either 10 µg/ml biotin-labeled Env Bal gp120 protein or biotin-labeled polyclonal goat anti-mouse IgG Ab. After incubation for 2 h at RT, the plate was washed and 100 µl of ALP-conjugated streptavidin (1∶1000 dilution in PBS) were added, then the plate was incubated at RT for 45 min. One hundred microliter of substrate was added and incubated at RT for 10 min. Plate was then washed extensively with water and air dried. Spots were counted using ChampSpot III Elispot Analysis System (Beijing Sage Creation).

### Cytokine Secretion in the Cultured Supernatant of Splenocytes

A half million of splenocytes per well in a 96-well plate were seeded and incubated at 37°C and 5% CO_2_ for 96 h, and the supernatants were harvested for detection IL-21 using the quantitative Mouse IL-21 Enzyme immunoassay Kit (Boster) according to the manufacture's protocol. The optical density values at 450 nm were read using the Model 550 Microplate Reader (Bio-Rad).

### Proliferation Activity of Splenocytes by the Env-peptide Stimulation

Proliferation activity of splenocytes was detected using a cell counting kit-8 (CCK-8, Dojindo Molecular Technologies). An 06044 wild-type homologus Env peptide that contains the potential T cell epitopes (ITLPCRIKQIINMWQ) of HIV-1 gp120 proteins was used as a stimulus in the proliferation assay and synthesized at GL Biochem. Ltd (Shanghai) [46]. A half million of splenocytes per well in a 96-well plate were seeded and maintained in the presence at 10 µg/ml or absence of the Env peptide. After incubation at 37°C and 5% CO_2_ for 96 h, cell viability was determined by CCK-8 assay and read at 450 nm using the Model 550 Microplate Reader (Bio-Rad). The proliferation index was calculated as OD450 values of the splenocytes with the peptide stimulation/OD450 values of cell control.

### Flow Cytometry

To identify specific cell subsets, single cell suspension of splenocytes in ice-cold FACS buffer (2% FCS and 0.1% NaN_3_ in PBS) were stained with the antibodies detailed below. Antibodies used for flow cytometric staining include anti-CD3-FITC (clone 145-2C11), anti-CD4-APC/Cy7 (clone GK1.5), anti-PD-1-PE/Cy7 (clone 29F.1A12), anti-CD44-APC (clone IM7), anti-CD62L-FITC (clone MEL-14) from Biolegend; anti-CXCR5-biotin (clone 2G8), Streptavidin-PE from BD Biosciences Pharmingen and each corresponding isotype-matched control antibodies. Flow cytometry data were acquired on a flow cytometer (CantoII; BD Biosciences) running FACS Diva and analyzed with FlowJo software (version 10; Tree Star).

### Statistical Analysis

All statistical analyses and data graphs were conducted with Prism 5 software (GraphPad Prism version 5.01; GraphPad Software). The lines in all figures were expressed as mean in each group. Differences between the means of the two continuous variables were evaluated by Student's *t* test with a two-tailed 95% confidence interval, unless otherwise indicated. Pearson's correlation coefficient was employed to determine linear relations between two parameters. A probability value of *P*<0.05 was considered to be significant.

## Results

### The Specific Antibody Responses, Immunoglobulin Secretion and Plasma Cell Production

**Figure 1:**
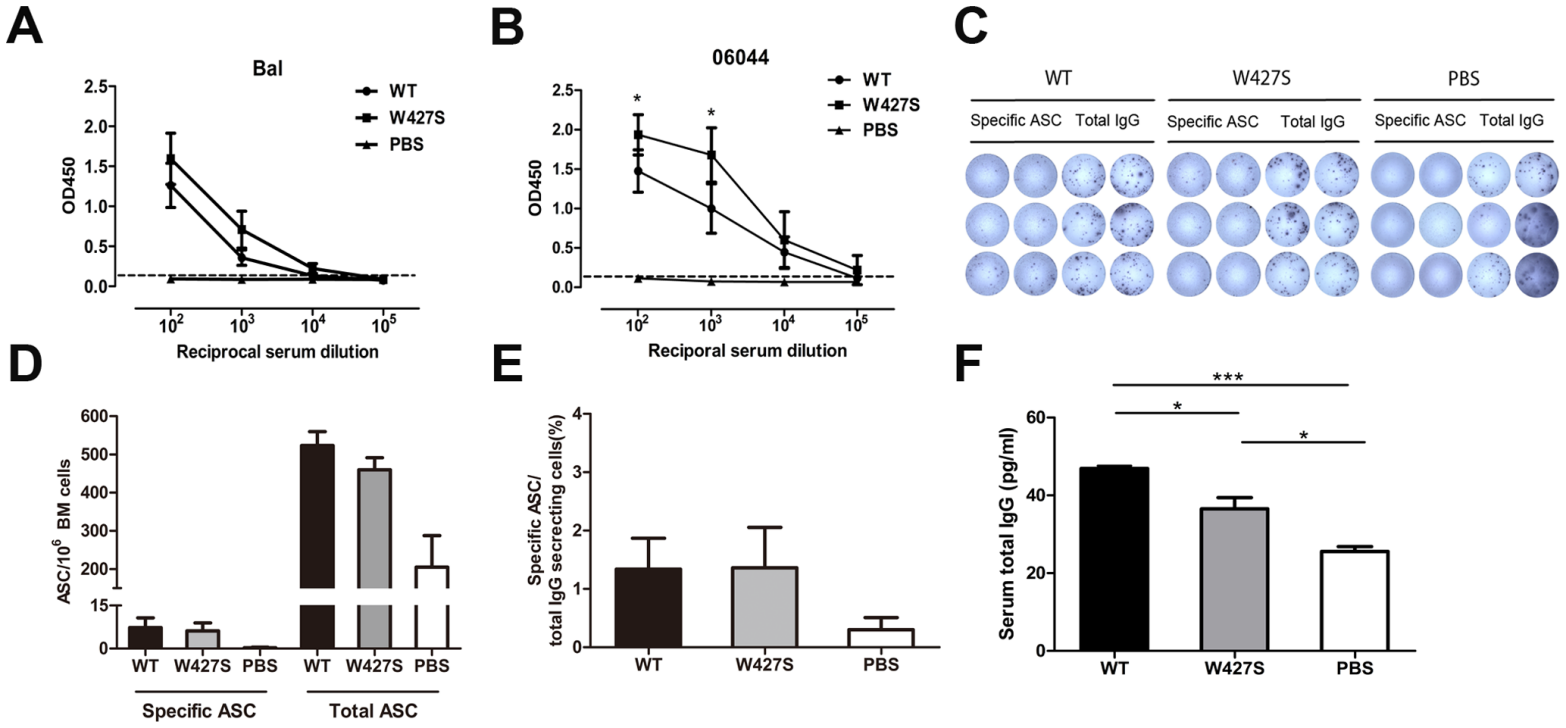
B cell responses after the immunization by HIV-1 Env gp120 and W427S mutant. (A–B) Specific binding antibodies in immunized mouse sera to Bal gp120 (A) and HIV-1 06044 gp120 (B) were detected by ELISA and shown as OD450 values at reciprocal serum dilutions. (C–E) HIV-1 Env-specific antibody-secreting cells (ASCs) and total IgG-secreting cells (SCs) were detected by B cell ELISPOT assay. The BM cells were isolated at day 14 after final immunization and added as duplicate in 96-well plate. Biotin-labeled HIV-1 Bal gp120 protein and biotin-labeled anti-mouse IgG antibodies were used for detection of specific ASC and total IgG SCs, respectively. (F) Total IgG levels in immunized mouse sera were measured by quantitative ELISA. WT, wild-type gp120. Data were shown as mean ± SEM. Asterisks indicate statistical significance (* *P*<0.05, *** *P*<0.001).

We firstly examined the specific antibody titers in the sera of mice immunized with the Envs. We found that the mutant W427S gp120 elicited higher specific antibody titers against HIV-1 Bal gp120 compared with the wild-type gp120 though there is no statistically significance (Fig. 1 A). For the homologues HIV-1 06044 gp120 protein binding, the mutant W427S gp120 elicited significantly higher specific antibody titers compared with the wild-type gp120 at dilution 1∶100 (*P = *0.0481) and 1∶1000 (*P = *0.0267) (Fig. 1 B). The endpoint titers of the specific binding Abs from the W427S and wild-type gp120 immunized mice were 3.87 and 3.64 to HIV-1 Bal gp120 protein, and 4.65 and 4.81 to HIV-1 06044 wild-type gp120 protein (reciprocal log10 dilution), respectively

Subsequently, we examined antibody-secreting cells (ASC) in bone marrow (BM) of the immunized mice (Fig. 1 C–E). We found a slightly higher frequency of total IgG ASCs in the wild-type gp120 immunized mice than that in W427S group, though no statistically significance was observed (Fig. 1 D). However, the wild-type gp120 did not induce higher specific ASC in BM compared with W427S (Fig. 1 E).

Accordingly, the wild-type gp120 immunized mice also displayed higher serum IgG concentrations than the W427S immunized mice (*P* = 0.0132). And, both wild-type and W427S groups showed higher serum IgG levels than the adjuvant control (*P*<0.001 and *P = *0.0135) (Fig. 1 F).

**Figure 2:**
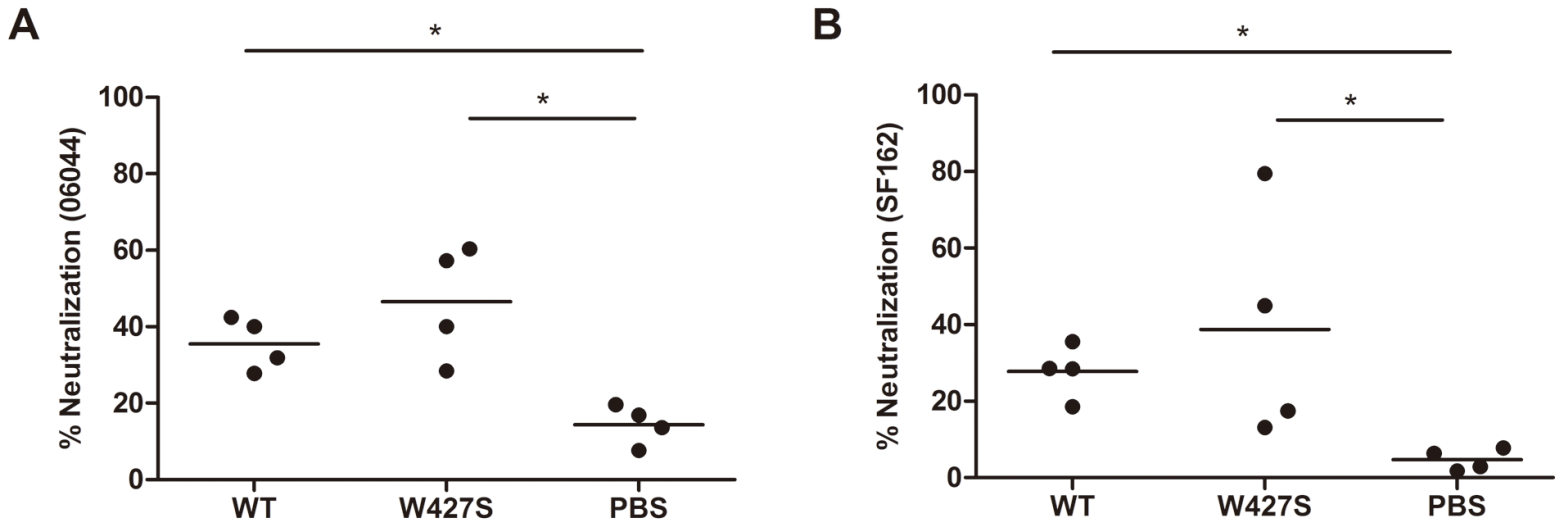
Neutralizing activities of immunized mouse sera. Neutralization assays were performed against homologous strain 06044 wild-type (A) and SF162 (B) Env-pseudoviruses. The results were expressed as neutralization percentage and the lines of mean neutralization percentage in each group were shown. Asterisks indicate statistical significance (* *P* <0.05).

However, wild-type gp120 did not elicit apparent neutralizing antibodies against either HIV-1 tier 1 strain SF162 or the homologous strain 06044. On contrary, W427S gp120 elicited neutralizing antibodies in 2 of 4 animals (neutralization percentages were equal or higher than 50%) against either 06044 or SF162 (Fig. 2 A, B). These results indicated that the W427S induced not only higher specific binding antibodies, but also the neutralizing activity.

### Tfh Cell Responses and IL-21 Secretion by Splenocytes after the Immunization

**Figure 3:**
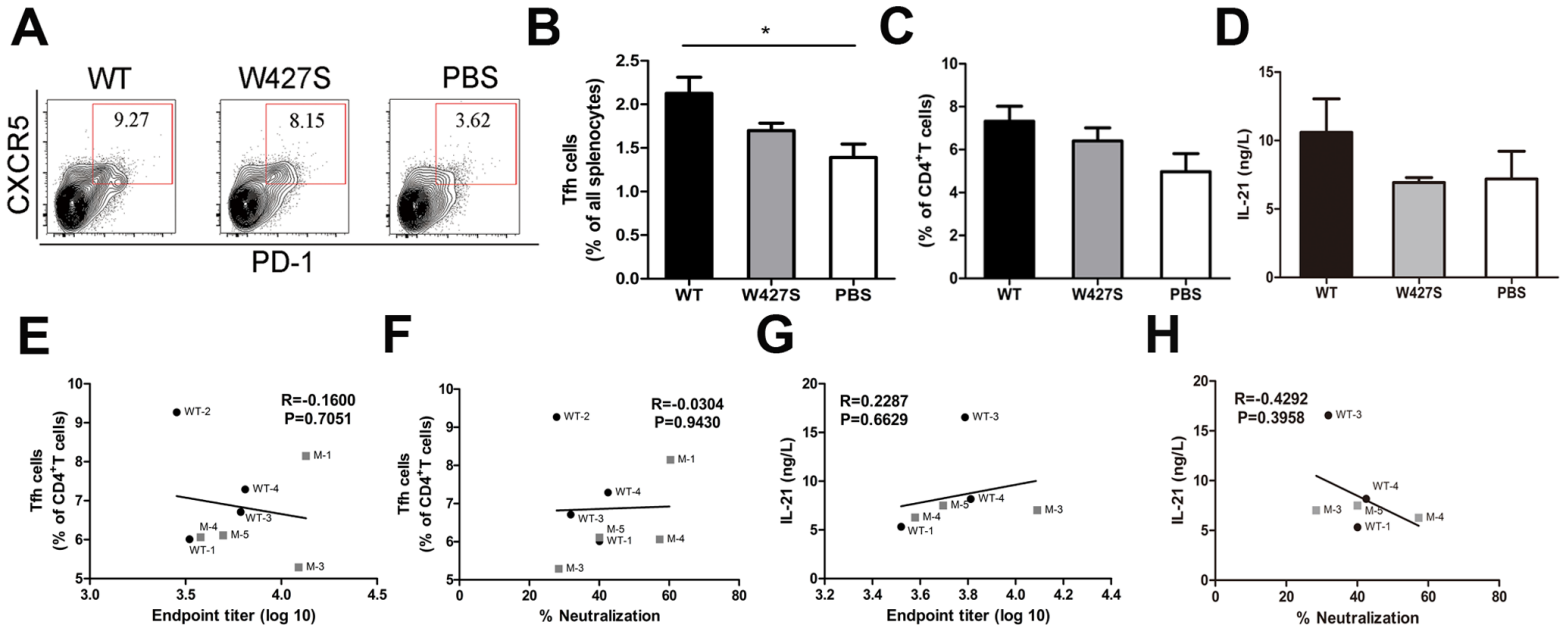
The frequency and function of Tfh cells after immunization. (A–C) Flow cytometry contour plots of splenocytes obtained at day 14 after final immunization (A) and numbers adjacent to outlined areas indicated the frequency (%) CXCR5^hi^PD-1^hi^ cells of total splenic lymphocytes (B) or CD4 T cell (C). (D) The secretory levels of IL-21 in the culture supernatants of splenocytes from immunized mice were detected by quantitative ELISA. (E) Correlation between Tfh frequency and endpoint titer of serum binding antibody. (F) Correlation between Tfh frequency and neutralization percentage. (G) Correlation between the secretory levels of IL-21 and endpoint titer of serum binding antibody. (H) Correlation between the secretory levels of IL-21 and neutralization percentage. R, correlation coefficient. Data were shown as mean ± SEM. WT depict the mice from 06044 WT group and M depict the mice from W427S group.

Helper CD4^+^ T cells are essential for differentiation of B cells and long-lived plasma cells [47]. Recently, it was reported that the expansion of HIV-specific Tfh cells occurred in HIV-1 infected patients [33], [34]. However, the Tfh cell functions in HIV-1 Env immunization have not been known. To clarify the contribution of Tfh cells in the induction of specific Abs, we evaluated the frequency and function of Tfh cells in the gp120-immunized mouse spleens (Fig. 3 A, B and C; S1 Fig.). We found a significant increase of the Tfh frequency in the all splenocytes after the immunization with the wild-type gp120 compared with the W427S gp120 (*P* = 0.0822). Moreover, the higher frequency of Tfh cells in the CD4^+^ T cell population in gp120 groups were observed compared with that in the adjuvant group (*P* = 0.0758). Furthermore, the concentrations of IL-21 in the splenocyte culture supernatants in the wild-type gp120 immunized mice were higher than those in W427S group and PBS group though no statistically significance was observed (Fig. 3 D).

We have known that Tfh cells are essential for helping production of antigen specific Abs, especially, nAbs. However, the wide-type gp120 immunization caused higher Tfh number, IL-21 secretion and lower specific Abs, we considered that not all Tfh subsets were involved in the induction of the specific Abs. Indeed, we found that both Tfh frequency and IL-21 concentrations were correlated with neither the binding antibody amounts nor the neutralization ability of the sera (Fig. 3 E-H).

### Splenocyte Proliferation to the Stimulation of A Gp120 Peptide

**Figure 4:**
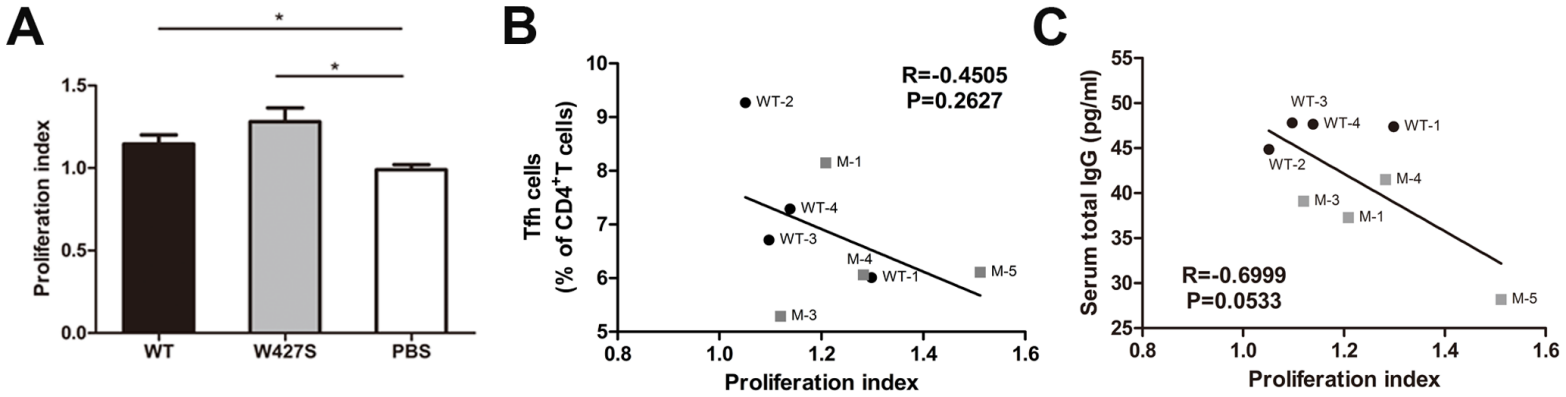
The specific proliferation activity of splenocytes following immunization. Splenocytes were collected at day 14 post-final immunization and cultured with 06044 wild-type homologous Env peptide for 4 days. (A) The peptide-specific proliferation was analyzed by CCK-8 assay and results were shown as proliferation index (OD450 values of the splenocytes with the peptide stimulation/OD450 values of cell control). (B) Correlation between the proliferation index and Tfh frequency. (C) Correlation between the proliferation index and serum total IgG concentrations was shown. R, correlation coefficient. Data were shown as mean ± SEM. Asterisks indicate statistical significance (* *P*<0.05). WT depict the mice from 06044 WT group and M depict the mice from W427S group.

We next thought if the higher ability of specific Ab induction by the W427S immunization was attributed to a stronger T cell proliferation against the specific epitopes. Therefore, we evaluated the proliferation of splenocytes stimulated by Env peptide that contains the potential T cell epitopes of HIV-1 gp120 proteins. We found that both gp120 immunization groups showed antigen-specific proliferations (proliferation indexes>1) compared with the adjuvant control. And, a slightly higher proliferation in W427S group compared with that in the wide-type group was also observed (*P* = 0.2262) (Fig. 4 A). However, there was a tendency that the proliferation of splenocytes was negatively correlated with Tfh amounts (R = −0.4505, *P* = 0.2627) (Fig. 4 B) and the serum IgG concentrations (R = −0.6999, *P* = 0.0533) (Fig. 4 C). Above findings suggested that W427S immunization caused stronger T cell proliferation but the proliferation of splenocytes is associated inversely with Tfh amounts and serum IgG concentrations.

### The Responses of Memory T (Tm) Cells after the Immunization

Since there was a stronger specific splenocyte proliferation and Ab response in the W427S gp120 immunized mice, we further assessed the memory T cells to explore the association of recall responses and the specific Ab production.

**Figure 5:**
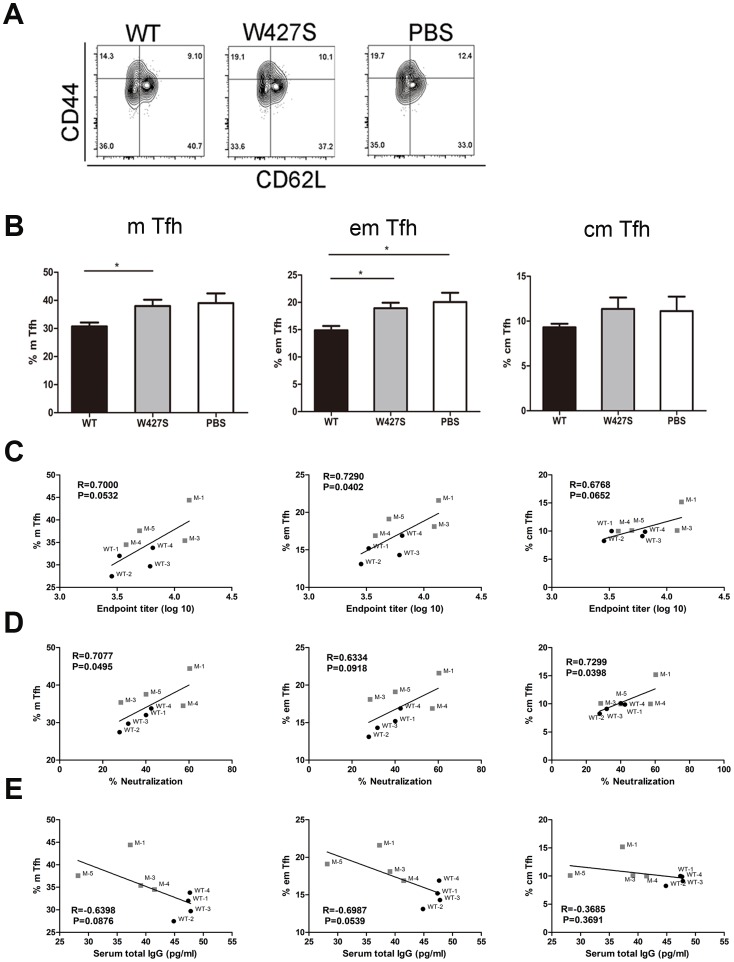
The frequency of memory Tfh (m Tfh) cells. (A, B) Flow cytometry contour plots of splenocytes obtained at day 14 after final immunization (A), and the frequencies (%) of m Tfh (CD4^+^CXCR5^hi^CD44^+^), effector memory Tfh (em Tfh, CD4^+^CXCR5^hi^CD44^+^CD62L^-^) and central memory Tfh (cm Tfh, CD4^+^CXCR5^hi^CD44^+^CD62L^+^) in the CD4^+^ T cell population were analyzed (B). Correlations of endpoint titer (C), neutralization percentage (D) and serum total IgG (E) with frequencies of m Tfh, em Tfh or cm Tfh were analyzed. R, correlation coefficient. Data were shown as mean ± SEM. Asterisks indicate statistical significance (* *P*<0.05). WT depict the mice from 06044 WT group and M depict the mice from W427S group.

We firstly examined the memory phenotype of CXCR5^+^CD4^+^ T cells which have been found as helper cells to B cells, termed as either pre-Tfh or Tfh [48]. We found that there were lower frequency of memory Tfh cells in the CD4^+^ T cell population of the wild-type gp120 immunized mice compared with that in the adjuvant control group (*P* = 0.0667), including both central memory Tfh (cm Tfh) (*P* = 0.3192) and effector memory Tfh cells (em Tfh) (*P* = 0.0332). However, the W427S gp120 immunization induced higher number of memory Tfh cells compared with wild-type gp120 immunization (*P* = 0.0331) (em Tfh cells *P* = 0.0194 and cm Tfh cells *P* = 0.1791) (Fig. 5 A, B; S1 Fig.). Correlation analysis indicated that the frequency of the memory Tfh cells were positively correlated with the specific binding Ab titers and the neutralization activities (Fig. 5 C and D). On the other hand, the memory Tfh frequency was negatively correlated with the serum IgG concentrations (Fig. 5 E).

Moreover, the frequency of the memory CD4^+^ T cells in all splenocytes of W427S gp120 immunized mice was also higher than that from wide-type gp120 immunized mice though no statistically significance was observed (S1 and S2 Figs.). And, the frequency of the memory CD4^+^ T cells was positively correlated with the specific binding Ab titers and the neutralization activities (S2 Fig.). Again, the memory CD4^+^ T cell frequencies were negatively correlated with the serum IgG concentrations (S2 Fig.).

## Discussion

In the present study, we modified the envelope of an HIV-1 primary R5 strain by substitution of 427W with S and immunized BALB/c mice using the DNA prime-protein boost strategy. We tried to clarify whether the modification affects the ability of the Env in eliciting nAb, and the possible mechanisms. We used the HIV-1 strain 06044 because the serum of 06044-infected individual exhibited a broad neutralization and its Env-pseudovirus showed high sensitivity to the bnAb b12 and the sera of HIV-1 CRF07_B/C as well as CRF01_A/E infected individuals (unpublished data).

In recent years, since some bnAbs targeting to CD4BS were found, the CD4BS has been used as the main target of HIV-1 vaccine design. However, the CD4BS in the context of the functional Env is poorly immunogenic [47]. The complete CD4BS is a discontinuous determinant composed of residues distributing over six structural segments of gp120: (1) the V1/V2 stem, (2) loop LD, (3) the β15-α3 excursion from the gp120 surface, (4) the β20-β21 hairpin, (5) the β23 strand, and (6) the β24-α5 connection loop [49]. Eight CD4-contacting amino acid residues are clustered in the linear stretch of residues 421–433 which located in the β20–β21 bridging sheet region, corresponding to 33% of the overall CD4BS [50]. This region includes the contiguous 425–430 region and constitutes the critical core of the CD4BS (CD4BS core) [40].

It has been found that the CD4BS core overlaps the B cell superantigen site of gp120 [40], [51]. Like other B cell superantigens, gp120 is recognized non-covalently by the framework regions (FRs) of pre-immune antibodies produced without prior exposure to HIV Env [52]–[54]. We designed a mutant of gp120, W427S, which has been known as the CD4-binding deficient and CD4BS-antibody-binding competent [11], [12]. We tried to reduce the superantigen effect of the CD4BS core maintaining the well exposed gp120 CD4BS.

We found that wild-type gp120 can induce more non-specific antibody-secreting plasma cells, higher serum IgG secretion, more Tfh cells. However, the modified W427S gp120 elicited higher levels of specific binding antibodies as well as nAbs though it produces less Tfh cells. Furthermore, higher Tfh cell frequency does not correlate to the specific binding Abs or nAbs indicating that the wild-type gp120 induced some non-specific Tfh that did not contribute to the production of specific Abs but the modification of gp120 may reduce the non-specific Ab production through reducing redundant Tfh cell frequency.

In chronic HIV-1 infection, both non-virus-specific and virus-specific Tfh cells are significantly expanded and the infected individuals rarely develop functional Tfh cells [33], [34]. Moreover, Tfh cells expansion has been also found in simian immunodeficiency virus (SIV) infections [35], [36]. The significance of the expansion of Tfh cells in HIV-1 infection in developing nAbs is still unclear.

In the context of immunization using HIV-1 immunogens, the Tfh responses as well as functions have not been studied extensively. Hollister K et al have found that the immunization with HIV-1 gp120 can also cause Tfh cell differentiation and GC B cells responses, elicit antigen-specific antibodies, but GC reaction may limit antigen-specific IgG secretion [37]. And, a viral superantigen may induce strong GC response [55]. Our findings also suggested that the modification of gp120 may decrease the limitation on specific Ab secretion by the strong Tfh response in the immunization with wild-type HIV-1 gp120.

We may also predict that a better immune memory was induced by the modified gp120 because this gp120 mutant immunization led to the recovery of memory Tfh numbers (including the central and the effector memory Tfh) and stronger T cell proliferation. Moreover, memory Tfh frequency is positively correlated with specific Abs, neutralization activities, proliferation of the splenocytes.

However, to date, there are lacking data regarding the mechanisms for Tfh memory. It has been known that central memory T cells are predominant in CD4^+^ T cells in blood and can rest for a long time in the absence of cognate antigens [12]. The central memory T cells have high level of STAT5 phosphorylation and possess an ability to self-renew [56]. And, the central memory T cells may tend to respond to viral and bacterial infections and also to cancers, stronger compared to the effector memory T cells [57], [58]. During cognate antigen stimulation, the central memory T cells readily proliferate and differentiate into the effector T cells to help B cell differentiation; while the effector T cells display effector functions immediately. Thus, the positive correlation between neutralization activity and memory T cell number implied that more memory T cell expansion resulted in more production of nAbs in the recall responses.

The frequency of memory B cells was similar among immunized animals with wide-type or W427S mutant gp120 (data not shown). Such a phenomenon was also observed in the immunized macaques comparing to B cell responses induced by influenza virus hemagglutinin proteins and HIV-1 Env [59]. More works are required to understand the mechanisms of B cell memory responses induced by HIV Env and other ideal immunogens.

To develop a prophylactic vaccine, the key point is to induce nAbs like other FDA-approved vaccines against infectious agents. One major hurdle in HIV-1 vaccine development is high variability that hampers the eliciting bnAbs against the enormous diversity of global circulating isolates [60], [61]. However, the results of the HIV-1 vaccine clinical trial, RV144 in Thailand, showed 31.2% prevention of HIV-1 infection [7], revealed that the protection is caused mainly by the direct binding Abs to the first and second variable loops (V1/V2) and first conserved region (C1) of Env [8], [62]. These results lead us to accept a concept that binding Abs, at least, to V1/V2 and C1 regions of gp120, might be also necessary. Recently, Carbonetti S and colleagues found that the soluble HIV-1 envelope immunogens derived from an elite neutralizer elicited cross-reacted Abs to V1V2 and induced binding Abs with low potency [63]. In our study, we found that W427S gp120 induced better nAb responses and higher levels of gp120-specific binding Abs compared with its wild-type counterpart. This implies a benefit for the design of new immunogens. Thus, strengthening the CD4BS-specific antibody induction, reducing its superantigen effect, and increasing productions of memory T cells may need to be considered.

## Supporting Information

S1 Fig **Gating strategies for T cell subsets from splenocytes.** Spleens were harvested from immunized mice at day 14 after final immunization. T cell subsets were defined as: Tfh cells (CD3^+^CD4^+^CXCR5^hi^PD-1^hi^) (A), memory Tfh cells (m Tfh, CD4^+^CXCR5^hi^CD44^+^), central memory Tfh cells (cm Tfh, CD4^+^CXCR5^hi^CD44^+^CD62L^+^), effector memory Tfh cells (em Tfh, CD4^+^CXCR5^hi^CD44^+^CD62L^-^) (B) and memory CD4^+^ T cells (m CD4^+^ T, CD4^+^CD44^+^) (C).
(TIF)

S2 Fig **Characterizations of memory CD4^+^ T cells.** (A) The frequencies (%) of memory CD4^+^ T cells (m CD4^+^ T, CD4^+^CD44^+^), effector memory CD4^+^ T cells (em CD4^+^ T, CD4^+^ CD44^+^CD62L^-^) and central memory CD4^+^ T cells (cm CD4^+^ T, CD4^+^CD44^+^CD62L^+^) were analyzed. Correlations of endpoint titer (B), neutralization percentage (C) and serum total IgG (D) with the frequencies of m CD4^+^ T cells, em CD4^+^ T cells or cm CD4^+^ T cells were shown. R, correlation coefficient. Data were shown as mean ± SEM. WT depict the mice from 06044 WT group and M depict the mice from W427S group.
(TIF)
