# Supplementary figures and images for: An HIV-1 Envelope Immunogen with W427S Mutation in CD4 Binding Site Induced More T Follicular Helper Memory Cells and Reduced Non-Specific Antibody Responses
Source: PLoS One. 2014 Dec 29;9(12):e115047. doi: 10.1371/journal.pone.0115047 (PMC4278894; doi:10.1371/journal.pone.0115047)

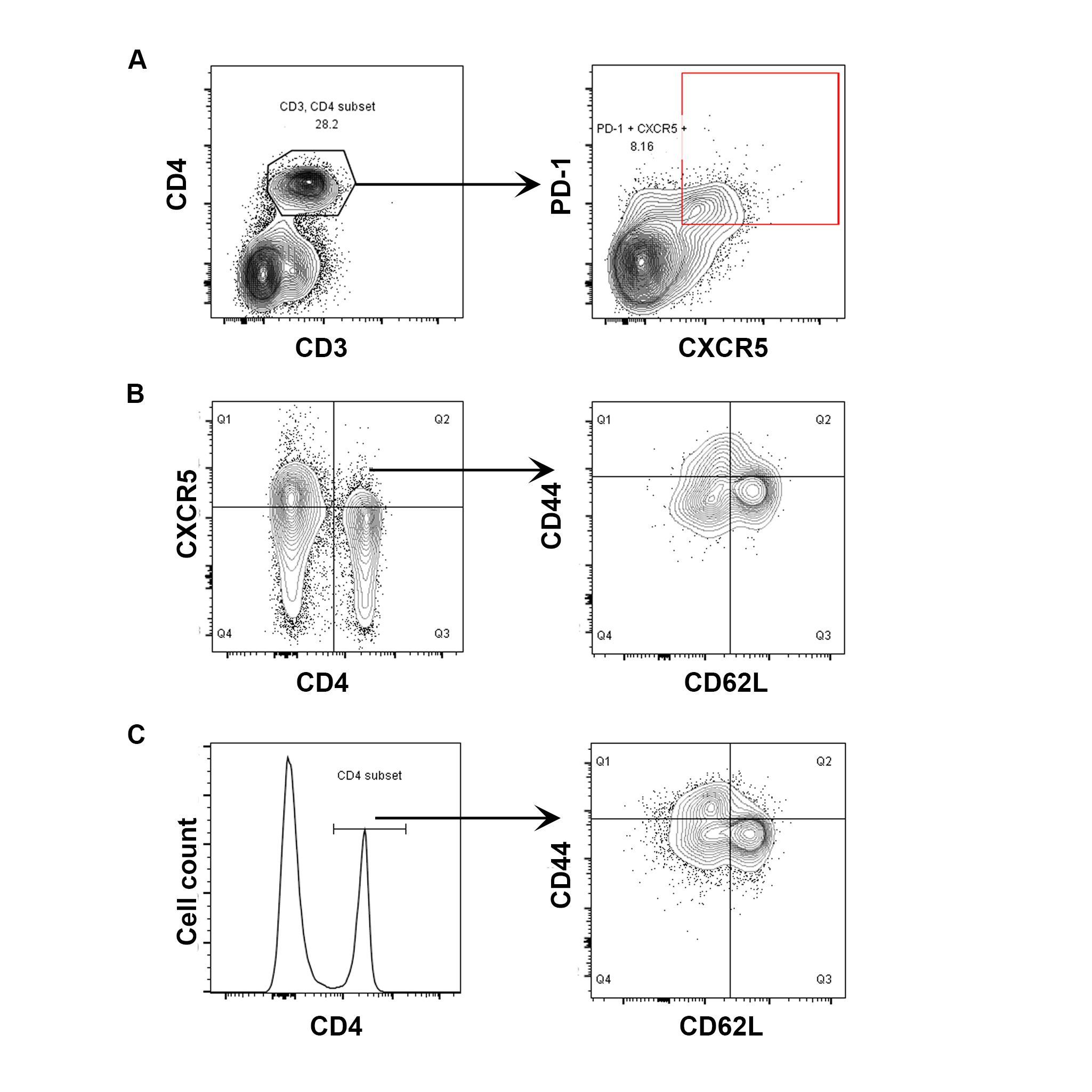

Supplement: S1 Fig — Gating strategies for T cell subsets from splenocytes. Spleens were harvested from immunized mice at day 14 after final immunization. T cell subsets were defined as: Tfh cells (CD3+CD4+CXCR5hiPD-1hi) (A), memory Tfh cells (m Tfh, CD4+CXCR5hiCD44+), central memory Tfh cells (cm Tfh, CD4+CXCR5hiCD44+CD62L+), effector memory Tfh cells (em Tfh, CD4+CXCR5hiCD44+CD62L-) (B) and memory CD4+ T cells (m CD4+ T, CD4+CD44+) (C). (TIF) [file pone.0115047.s001.tif]

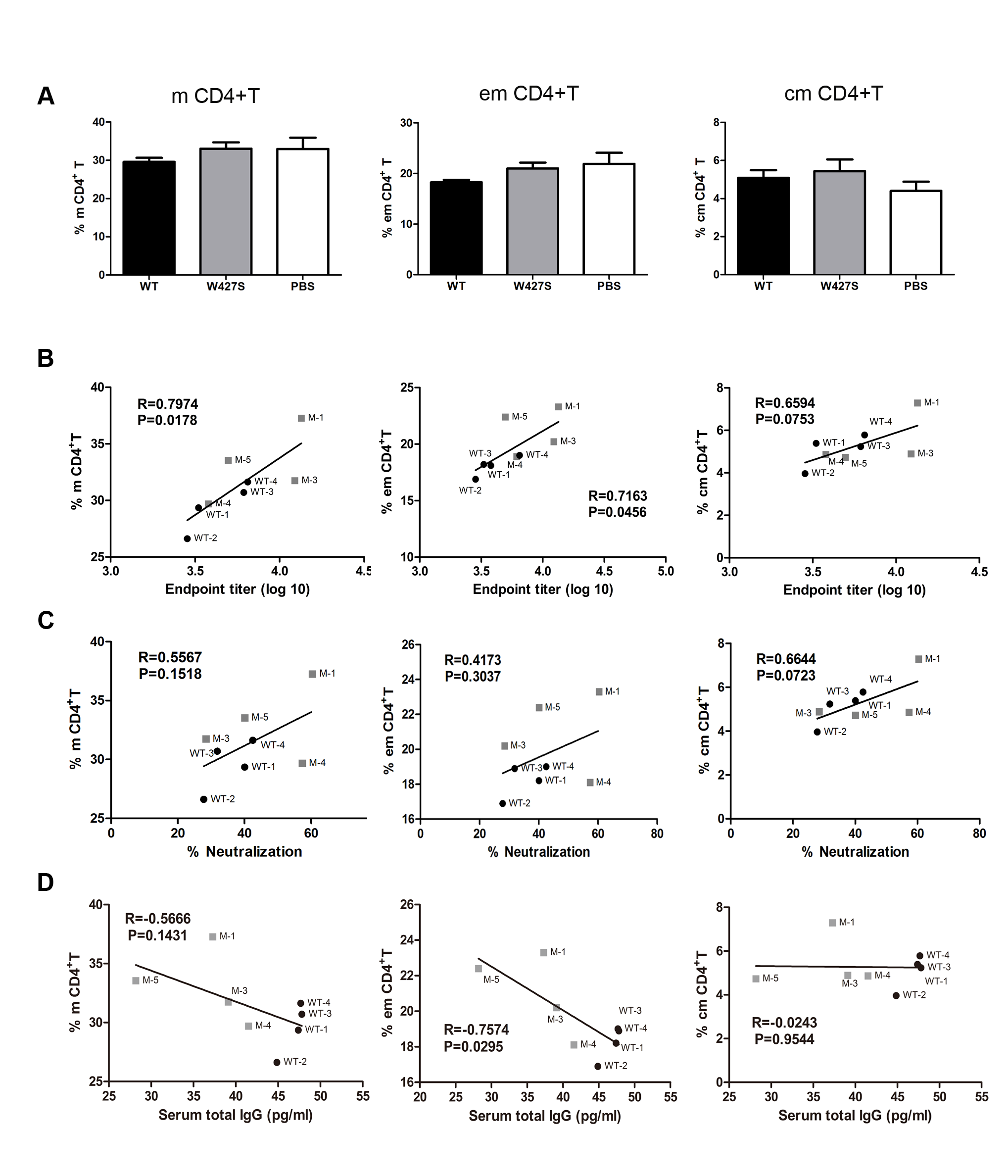

Supplement: S2 Fig — Characterizations of memory CD4+ T cells. (A) The frequencies (%) of memory CD4+ T cells (m CD4+ T, CD4+CD44+), effector memory CD4+ T cells (em CD4+ T, CD4+ CD44+CD62L-) and central memory CD4+ T cells (cm CD4+ T, CD4+CD44+CD62L+) were analyzed. Correlations of endpoint titer (B), neutralization percentage (C) and serum total IgG (D) with the frequencies of m CD4+ T cells, em CD4+ T cells or cm CD4+ T cells were shown. R, correlation coefficient. Data were shown as mean ± SEM. WT depict the mice from 06044 WT group and M depict the mice from W427S group. (TIF) [file pone.0115047.s002.tif]
